# Diaqua­bis­(5-methyl­pyrazine-2-carboxyl­ato-κ^2^ *N* ^1^,*O* ^2^)cadmium

**DOI:** 10.1107/S1600536811035045

**Published:** 2011-09-14

**Authors:** Joselyn Albanez, Iván Brito, Alejandro Cárdenas, Matías López-Rodríguez

**Affiliations:** a Departamento de Química, Facultad de Ciencias Básicas, Universidad de Antofagasta, Casilla 170, Antofagasta, Chile; b Departamento de Física, Facultad de Ciencias Básicas, Universidad de Antofagasta, Casilla 170, Antofagasta, Chile; c Instituto de Bio-Orgánica ‘Antonio González’, Universidad de La Laguna, Astrofísico Francisco Sánchez N°2, La Laguna, Tenerife, Spain

## Abstract

In the title compound, [Cd(C_6_H_5_N_2_O_2_)_2_(H_2_O)_2_], the Cd^II^ ion is coordinated in a severely distorted octa­hedral geometry. The N atoms are *cis* to each other, while the water O atoms and ligand O atoms are mutually *trans*. The crystal structure is stabilized by a network of O—H⋯O, O—H⋯N and C—H⋯O hydrogen bonds and π–π stacking inter­actions [centroid–centroid distances = 3.730 (3) and 3.652 (3) Å] between the 5-methyl­pyrazine-2-carboxyl­ate ligands. The structure is isotypic with the manganese analog.

## Related literature

For background to coordination chemistry, see: Blake *et al.* (1999 ▶); Brito *et al.* (2011 ▶). For the isotypic Mn compound see: Chapman *et al.* (2002 ▶). For similar compounds of the type [*M*(C_6_H_5_N_2_O_2_)_2_(H_2_O)_2_, where *M* = Fe^II^, Co^II^, Zn^II^, Ni^II^] see: Fan *et al.* (2007*a* ▶,*b* ▶, 2009 ▶); Shang *et al.* (2007 ▶).
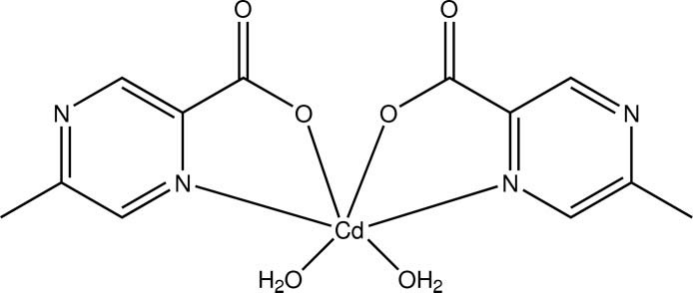


## Experimental

### 

#### Crystal data

[Cd(C_6_H_5_N_2_O_2_)_2_(H_2_O)_2_]
*M* *_r_* = 422.67
Triclinic, 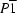

*a* = 7.2900 (15) Å
*b* = 7.5320 (15) Å
*c* = 14.090 (3) Å
α = 87.31 (3)°
β = 81.36 (3)°
γ = 80.78 (3)°
*V* = 754.8 (3) Å^3^
*Z* = 2
Mo *K*α radiation
μ = 1.48 mm^−1^
*T* = 293 K
0.44 × 0.40 × 0.22 mm

#### Data collection

Oxford Diffraction CCD area-detector diffractometer
Absorption correction: multi-scan (*MULABS*; Spek, 2009 ▶; Blessing, 1995 ▶) *T*_min_ = 0.561, *T*_max_ = 0.736
5896 measured reflections
3530 independent reflections
2778 reflections with *I* > 2σ(*I*)
*R*_int_ = 0.061

#### Refinement

*R*[*F* ^2^ > 2σ(*F* ^2^)] = 0.058
*wR*(*F* ^2^) = 0.159
*S* = 1.06
3530 reflections
226 parameters
H atoms treated by a mixture of independent and constrained refinement
Δρ_max_ = 2.23 e Å^−3^
Δρ_min_ = −1.11 e Å^−3^

### 

Data collection: *CrysAlis PRO* (Agilent, 2010 ▶); cell refinement: *CrysAlis PRO*; data reduction: *CrysAlis PRO*; program(s) used to solve structure: *SHELXS97* (Sheldrick, 2008 ▶); program(s) used to refine structure: *SHELXL97* (Sheldrick, 2008 ▶); molecular graphics: *OLEX2* (Dolomanov *et al.*, 2009 ▶) and *PLATON* (Spek, 2009 ▶); software used to prepare material for publication: *publCIF* (Westrip, 2010 ▶) and *WinGX* (Farrugia, 1999 ▶).

## Supplementary Material

Crystal structure: contains datablock(s) I, global. DOI: 10.1107/S1600536811035045/bt5630sup1.cif

Structure factors: contains datablock(s) I. DOI: 10.1107/S1600536811035045/bt5630Isup2.hkl

Additional supplementary materials:  crystallographic information; 3D view; checkCIF report

## Figures and Tables

**Table:** 

Cd1—O1	2.245 (4)
Cd1—O2	2.269 (4)
Cd1—O4	2.278 (5)
Cd1—O3	2.283 (5)
Cd1—N3	2.361 (5)
Cd1—N1	2.370 (5)

**Table:** 

O2—Cd1—O4	154.86 (19)
O1—Cd1—O3	161.4 (2)
O2—Cd1—N3	71.25 (16)
O3—Cd1—N3	89.0 (2)
O1—Cd1—N1	72.18 (16)
N3—Cd1—N1	177.55 (14)

**Table 2:** Hydrogen-bond geometry (Å, °)

*D*—H⋯*A*	*D*—H	H⋯*A*	*D*⋯*A*	*D*—H⋯*A*
O3—H3*A*⋯N4^i^	0.75 (6)	2.12 (6)	2.875 (9)	173 (6)
O3—H3*B*⋯O5^ii^	0.73 (10)	2.08 (10)	2.740 (8)	150 (10)
O4—H4*A*⋯N2^iii^	0.84 (8)	2.04 (8)	2.852 (8)	164 (8)
O4—H4*B*⋯O6^iv^	0.93 (11)	1.99 (10)	2.811 (8)	146 (8)
C4—H4⋯O5^ii^	0.93	2.29	3.211 (7)	169

Symmetry codes: (i) 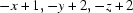; (ii) 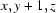; (iii) 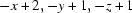; (iv) 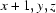.

**Fig. 1.**
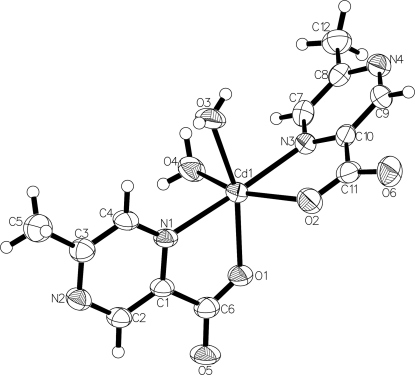
The molecule of (I), showing the atom-labelling scheme. Displacement ellipsoids are drawn at the 30% probability level. H atoms are represented by circles of arbitrary radius.

**Fig. 2.**
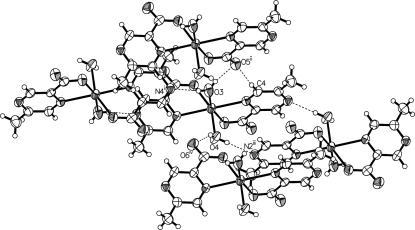
A view of title compound showing the hydrogen bonds and are indicated by dashed lines. [Symmetry codes: (i) -x + 1, -y + 2, -z + 2; (ii) x, y + 1, z; (iii) -x + 2, -y + 1, -z + 1; (iv) x + 1, y, z].

**Fig. 3.**
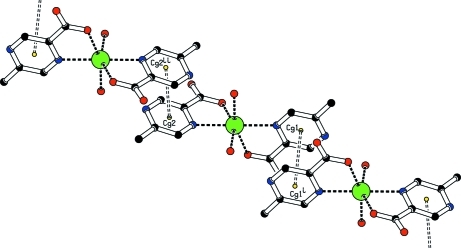
A partial packing diagram (I), showing molecules stacked view along ab plane. Ring centroids (Cg1 and Cg2 for the 2-methylpyrazine rings) involved in the π-π interactions are joined by dashed lines. [Symmetry codes: (i) -x + 2, -y + 1, -z + 1; (ii) -x + 1, -y + 2, -z + 2]

## supplementary crystallographic information

### Comment

The design of polymeric organic-inorganic materials with novel topologies and structural motifs is of current interest in the field of coordination chemistry, (Blake *et al.*, 1999). This paper forms part of our continuing study of the synthesis, structural characterization and physical properties of coordination polymers (Brito *et al.*, 2011). The title compound was isolated during attempts to synthesize a mixed-ligand coordination polymer by a condensation reaction between the title compound and pyrazine. The crystal structure of the title compound, contains discrete mononuclear complex molecules in which Cd ^II^ ions, are chelated by two 2-methylpyrazine- 5-carboxylate ligands in a *trans*-*cis* mode and bonded by two water molecules. The coordination geometry around the Cd^II^ ion is severely distorted octahedral. The ligand chelation proceeds *via* its N,*O*-bonding group. The organic ligands are essentially planar [r.m.s. deviation 0.0765 (3) Å mean] and form a dihedral angle of 89.71 (18)°. The molecular structure is shown in Fig. 1 and relevant bond distances and angles for the Cd^II^ coordination octahedron are listed in Table 1. All molecular geometry parameters lie within the normal ranges, except the C1—C6 bond distance (1.532 (8) Å) which is longer than C*sp*^2^—C*sp*^2^ bond distance, possibly due to the coordination effect of the Cd atom. This effect is observed in Mn analog. The coordination geometry and geometric parameters of the title compound match closely those found in the analog compound (Chapman *et al.*, 2002).The crystal structure is stabilized by a network of O—H···O, O—H···N, C—H···O, hydrogen bonds, forming an infinite 3-D network, Fig. 2, Table 2, and π-π stacking interactions between the 5-methylpyrazine-2-carboxylate ligand, Fig. 3, Table 3.

### Experimental

5-methylpyrazine-2-carboxylic acid (1113 mg, 8.0 mmol) was added to 20 ml solution of NaOH (322 mg, 8.0 mmol) in water. The mixture was stirred for 30 minutes at room temperature. CdCl_2_ (739 mg, 4.0 mmol) was added slowly to the above solution and upon heating, a yellow precipitate was formed. After filtration the yellow material was washed several times with water and dried in air. The title compound was obtained by hydrothermal synthesis of a mixture of the yellow  precipitate (105.6 mg, 0.25 mmol) and pyrazine (40.0 mg, 0.5 mmol) in 6 ml H_2_O, in an acid digestion bomb, heated at 130 °C for 72 h. Suitable single crystals grew upon cooling of the solution to room temperature. C_12_H_14_CdN_4_O_6_; IR (KBr, cm^-1^): ν = 3427 s, 1631 s,1580 s, 1520 m, 1483 m, 1449 s, 1395 s, 1322 s, 1290 m, 1171 m, 1042 s, 871 s, 413 s.

### Refinement

Hydrogen atoms were located in a difference Fourier map but they were included in calculated positions [C—H = 0.93 - 0.96 Å and refined as riding [*U*_iso_(H) = 1.2*U*_eq_(C) and 1.5*U*_eq_(C) for methyl H atoms]. Water H atoms (H3A, H3B, H4A and H4B) were refined isotropically. The single-crystal used was curved and weakly diffracting, with only 82% of the reflections considered to be observed. However, this fact did not adversely affect the solution and refinement processes. The highest electron-density peak and the deepest hole are located 0.99 and 1.06 Å from atom Cd in the final difference Fourier.

### Crystal data

**Table:**

[Cd(C_6_H_5_N_2_O_2_)_2_(H_2_O)_2_]	*Z* = 2
*M**_r_* = 422.67	*F*(000) = 420
Triclinic, *P*1	*D*_x_ = 1.860 Mg m^−^^3^
Hall symbol: -P 1	Mo *K*α radiation, λ = 0.71073 Å
*a* = 7.2900 (15) Å	Cell parameters from 5011 reflections
*b* = 7.5320 (15) Å	θ = 3.4–29.7°
*c* = 14.090 (3) Å	µ = 1.48 mm^−^^1^
α = 87.31 (3)°	*T* = 293 K
β = 81.36 (3)°	Block, yellow
γ = 80.78 (3)°	0.44 × 0.40 × 0.22 mm
*V* = 754.8 (3) Å^3^	

### Data collection

**Table:**

Oxford Diffraction CCD area-detector diffractometer	3530 independent reflections
Radiation source: fine-focus sealed tube	2778 reflections with *I* > 2σ(*I*)
graphite	*R*_int_ = 0.061
ω scans	θ_max_ = 29.7°, θ_min_ = 3.4°
Absorption correction: multi-scan (*MULABS*; Spek, 2009; Blessing, 1995)	*h* = −8→9
*T*_min_ = 0.561, *T*_max_ = 0.736	*k* = −9→5
5896 measured reflections	*l* = −17→17

### Refinement

**Table:**

Refinement on *F*^2^	Primary atom site location: structure-invariant direct methods
Least-squares matrix: full	Secondary atom site location: difference Fourier map
*R*[*F*^2^ > 2σ(*F*^2^)] = 0.058	Hydrogen site location: inferred from neighbouring sites
*wR*(*F*^2^) = 0.159	H atoms treated by a mixture of independent and constrained refinement
*S* = 1.06	*w* = 1/[σ^2^(*F*_o_^2^) + (0.0751*P*)^2^] where *P* = (*F*_o_^2^ + 2*F*_c_^2^)/3
3530 reflections	(Δ/σ)_max_ = 0.002
226 parameters	Δρ_max_ = 2.23 e Å^−^^3^
0 restraints	Δρ_min_ = −1.11 e Å^−^^3^

### Special details

**Table:**

Geometry. All s.u.'s (except the s.u. in the dihedral angle between two l.s. planes) are estimated using the full covariance matrix. The cell s.u.'s are taken into account individually in the estimation of s.u.'s in distances, angles and torsion angles; correlations between s.u.'s in cell parameters are only used when they are defined by crystal symmetry. An approximate (isotropic) treatment of cell s.u.'s is used for estimating s.u.'s involving l.s. planes.
Refinement. Refinement of *F*^2^ against ALL reflections. The weighted *R*-factor *wR* and goodness of fit *S* are based on *F*^2^, conventional *R*-factors *R* are based on *F*, with *F* set to zero for negative *F*^2^. The threshold expression of *F*^2^ > 2σ(*F*^2^) is used only for calculating *R*-factors(gt) *etc*. and is not relevant to the choice of reflections for refinement. *R*-factors based on *F*^2^ are statistically about twice as large as those based on *F*, and *R*- factors based on ALL data will be even larger.

### Fractional atomic coordinates and isotropic or equivalent isotropic displacement parameters (Å^2^)

**Table:**

	*x*	*y*	*z*	*U*_iso_*/*U*_eq_	
Cd1	0.61134 (5)	0.67712 (5)	0.73978 (3)	0.03763 (19)	
O1	0.6127 (6)	0.3946 (6)	0.6951 (3)	0.0472 (11)	
O2	0.2944 (6)	0.7316 (6)	0.7793 (3)	0.0475 (10)	
O3	0.6360 (9)	0.9759 (7)	0.7328 (5)	0.0593 (14)	
H3A	0.625 (7)	1.019 (8)	0.781 (4)	0.020 (15)*	
H3B	0.607 (14)	1.024 (13)	0.690 (7)	0.09 (4)*	
O4	0.9122 (7)	0.6009 (8)	0.7706 (4)	0.0581 (13)	
H4A	0.995 (10)	0.560 (10)	0.726 (6)	0.06 (2)*	
H4B	0.943 (13)	0.705 (14)	0.793 (7)	0.10 (3)*	
O5	0.6558 (8)	0.2045 (6)	0.5758 (3)	0.0561 (12)	
O6	0.0540 (6)	0.8123 (8)	0.8930 (4)	0.0607 (13)	
N1	0.6959 (7)	0.6675 (6)	0.5708 (3)	0.0354 (10)	
N2	0.8477 (7)	0.5928 (7)	0.3815 (4)	0.0421 (12)	
N3	0.5370 (6)	0.6945 (6)	0.9084 (3)	0.0346 (10)	
N4	0.4226 (7)	0.8321 (7)	1.0909 (4)	0.0411 (12)	
C1	0.7138 (7)	0.5011 (7)	0.5376 (4)	0.0344 (12)	
C2	0.7863 (8)	0.4661 (8)	0.4434 (4)	0.0398 (13)	
H2	0.7933	0.3503	0.4215	0.048*	
C3	0.8288 (8)	0.7586 (8)	0.4138 (4)	0.0418 (13)	
C4	0.7530 (9)	0.7945 (8)	0.5084 (4)	0.0417 (13)	
H4	0.7412	0.9115	0.5295	0.050*	
C5	0.8947 (11)	0.9043 (10)	0.3470 (5)	0.0591 (18)	
H5A	0.9502	0.9833	0.3817	0.089*	
H5B	0.7898	0.9714	0.3207	0.089*	
H5C	0.9862	0.8512	0.2958	0.089*	
C6	0.6543 (8)	0.3540 (8)	0.6091 (5)	0.0400 (13)	
C7	0.6558 (8)	0.6841 (8)	0.9727 (4)	0.0405 (13)	
H7	0.7798	0.6306	0.9555	0.049*	
C8	0.6001 (8)	0.7502 (8)	1.0637 (4)	0.0392 (13)	
C9	0.3029 (8)	0.8382 (8)	1.0262 (4)	0.0425 (14)	
H9	0.1785	0.8906	1.0433	0.051*	
C10	0.3579 (7)	0.7703 (8)	0.9364 (4)	0.0365 (12)	
C11	0.2241 (8)	0.7717 (8)	0.8637 (5)	0.0424 (14)	
C12	0.7340 (10)	0.7352 (10)	1.1345 (5)	0.0532 (17)	
H12A	0.7154	0.8453	1.1688	0.080*	
H12B	0.8603	0.7131	1.1015	0.080*	
H12C	0.7127	0.6375	1.1789	0.080*	

### Atomic displacement parameters (Å^2^)

**Table:**

	*U*^11^	*U*^22^	*U*^33^	*U*^12^	*U*^13^	*U*^23^
Cd1	0.0420 (3)	0.0324 (3)	0.0377 (3)	−0.00852 (18)	0.00199 (18)	−0.00820 (19)
O1	0.069 (3)	0.033 (2)	0.041 (2)	−0.019 (2)	0.003 (2)	−0.0051 (19)
O2	0.042 (2)	0.058 (3)	0.042 (2)	−0.005 (2)	−0.0048 (18)	−0.010 (2)
O3	0.102 (4)	0.035 (3)	0.041 (3)	−0.021 (3)	−0.001 (3)	−0.011 (3)
O4	0.037 (3)	0.070 (4)	0.066 (3)	−0.003 (2)	−0.001 (2)	−0.032 (3)
O5	0.092 (4)	0.027 (2)	0.050 (3)	−0.017 (2)	−0.003 (2)	−0.008 (2)
O6	0.031 (2)	0.086 (4)	0.066 (3)	−0.009 (2)	−0.002 (2)	−0.015 (3)
N1	0.048 (3)	0.023 (2)	0.036 (3)	−0.0080 (19)	−0.004 (2)	−0.005 (2)
N2	0.048 (3)	0.035 (3)	0.042 (3)	−0.003 (2)	−0.002 (2)	−0.007 (2)
N3	0.034 (2)	0.033 (3)	0.036 (3)	−0.0069 (19)	0.0004 (19)	−0.005 (2)
N4	0.049 (3)	0.036 (3)	0.038 (3)	−0.008 (2)	−0.002 (2)	−0.004 (2)
C1	0.037 (3)	0.023 (3)	0.043 (3)	−0.003 (2)	−0.005 (2)	−0.008 (2)
C2	0.042 (3)	0.030 (3)	0.047 (4)	−0.004 (2)	−0.004 (2)	−0.011 (3)
C3	0.049 (3)	0.036 (3)	0.040 (3)	−0.007 (3)	−0.008 (3)	0.000 (3)
C4	0.057 (4)	0.023 (3)	0.044 (3)	−0.005 (2)	0.000 (3)	−0.007 (2)
C5	0.076 (5)	0.053 (4)	0.050 (4)	−0.017 (4)	−0.006 (3)	0.002 (3)
C6	0.043 (3)	0.030 (3)	0.047 (4)	−0.006 (2)	−0.006 (3)	−0.006 (3)
C7	0.032 (3)	0.044 (4)	0.044 (3)	−0.007 (2)	−0.001 (2)	0.002 (3)
C8	0.041 (3)	0.036 (3)	0.042 (3)	−0.014 (2)	−0.003 (2)	0.003 (3)
C9	0.036 (3)	0.041 (3)	0.047 (4)	−0.005 (2)	0.005 (2)	−0.012 (3)
C10	0.033 (3)	0.032 (3)	0.043 (3)	−0.010 (2)	0.005 (2)	−0.003 (2)
C11	0.038 (3)	0.035 (3)	0.055 (4)	−0.009 (2)	−0.004 (3)	−0.008 (3)
C12	0.060 (4)	0.055 (4)	0.050 (4)	−0.017 (3)	−0.016 (3)	−0.004 (3)

### Geometric parameters (Å, °)

**Table:**

Cd1—O1	2.245 (4)	N4—C9	1.347 (8)
Cd1—O2	2.269 (4)	N4—C8	1.349 (8)
Cd1—O4	2.278 (5)	C1—C2	1.374 (8)
Cd1—O3	2.283 (5)	C1—C6	1.532 (8)
Cd1—N3	2.361 (5)	C2—H2	0.9300
Cd1—N1	2.370 (5)	C3—C4	1.385 (8)
O1—C6	1.244 (7)	C3—C5	1.502 (9)
O2—C11	1.253 (8)	C4—H4	0.9300
O3—H3A	0.75 (6)	C5—H5A	0.9600
O3—H3B	0.73 (9)	C5—H5B	0.9600
O4—H4A	0.83 (8)	C5—H5C	0.9600
O4—H4B	0.93 (11)	C7—C8	1.376 (8)
O5—C6	1.238 (7)	C7—H7	0.9300
O6—C11	1.240 (7)	C8—C12	1.486 (8)
N1—C1	1.339 (7)	C9—C10	1.366 (8)
N1—C4	1.342 (7)	C9—H9	0.9300
N2—C3	1.329 (8)	C10—C11	1.516 (8)
N2—C2	1.346 (8)	C12—H12A	0.9600
N3—C7	1.335 (7)	C12—H12B	0.9600
N3—C10	1.347 (7)	C12—H12C	0.9600
			
O1—Cd1—O2	93.45 (17)	N2—C3—C4	120.1 (6)
O1—Cd1—O4	90.14 (19)	N2—C3—C5	119.0 (6)
O2—Cd1—O4	154.86 (19)	C4—C3—C5	120.9 (6)
O1—Cd1—O3	161.4 (2)	N1—C4—C3	122.7 (5)
O2—Cd1—O3	92.9 (2)	N1—C4—H4	118.6
O4—Cd1—O3	91.5 (2)	C3—C4—H4	118.6
O1—Cd1—N3	109.65 (16)	C3—C5—H5A	109.5
O2—Cd1—N3	71.25 (16)	C3—C5—H5B	109.5
O4—Cd1—N3	84.11 (18)	H5A—C5—H5B	109.5
O3—Cd1—N3	89.0 (2)	C3—C5—H5C	109.5
O1—Cd1—N1	72.18 (16)	H5A—C5—H5C	109.5
O2—Cd1—N1	110.48 (16)	H5B—C5—H5C	109.5
O4—Cd1—N1	94.30 (19)	O5—C6—O1	126.1 (6)
O3—Cd1—N1	89.2 (2)	O5—C6—C1	116.6 (6)
N3—Cd1—N1	177.55 (14)	O1—C6—C1	117.2 (5)
C6—O1—Cd1	120.4 (4)	N3—C7—C8	122.0 (5)
C11—O2—Cd1	119.5 (4)	N3—C7—H7	119.0
Cd1—O3—H3A	114 (4)	C8—C7—H7	119.0
Cd1—O3—H3B	115 (8)	N4—C8—C7	120.9 (5)
H3A—O3—H3B	124 (10)	N4—C8—C12	118.0 (6)
Cd1—O4—H4A	119 (5)	C7—C8—C12	121.1 (6)
Cd1—O4—H4B	105 (6)	N4—C9—C10	122.3 (5)
H4A—O4—H4B	109 (8)	N4—C9—H9	118.8
C1—N1—C4	116.9 (5)	C10—C9—H9	118.8
C1—N1—Cd1	112.5 (4)	N3—C10—C9	120.8 (5)
C4—N1—Cd1	129.8 (4)	N3—C10—C11	116.2 (5)
C3—N2—C2	117.3 (5)	C9—C10—C11	123.0 (5)
C7—N3—C10	117.3 (5)	O6—C11—O2	125.5 (6)
C7—N3—Cd1	127.7 (4)	O6—C11—C10	117.1 (6)
C10—N3—Cd1	112.4 (4)	O2—C11—C10	117.4 (5)
C9—N4—C8	116.6 (5)	C8—C12—H12A	109.5
N1—C1—C2	120.3 (5)	C8—C12—H12B	109.5
N1—C1—C6	117.3 (5)	H12A—C12—H12B	109.5
C2—C1—C6	122.4 (5)	C8—C12—H12C	109.5
N2—C2—C1	122.7 (6)	H12A—C12—H12C	109.5
N2—C2—H2	118.7	H12B—C12—H12C	109.5
C1—C2—H2	118.7		
			
O2—Cd1—O1—C6	110.9 (5)	C6—C1—C2—N2	−176.5 (5)
O4—Cd1—O1—C6	−94.0 (5)	C2—N2—C3—C4	1.6 (8)
O3—Cd1—O1—C6	1.2 (9)	C2—N2—C3—C5	−179.7 (5)
N3—Cd1—O1—C6	−177.8 (4)	C1—N1—C4—C3	−0.5 (8)
N1—Cd1—O1—C6	0.5 (4)	Cd1—N1—C4—C3	168.3 (4)
O1—Cd1—O2—C11	119.9 (5)	N2—C3—C4—N1	0.0 (9)
O4—Cd1—O2—C11	22.2 (7)	C5—C3—C4—N1	−178.7 (6)
O3—Cd1—O2—C11	−77.7 (5)	Cd1—O1—C6—O5	−178.3 (5)
N3—Cd1—O2—C11	10.3 (5)	Cd1—O1—C6—C1	3.2 (7)
N1—Cd1—O2—C11	−167.9 (4)	N1—C1—C6—O5	173.8 (5)
O1—Cd1—N1—C1	−4.4 (3)	C2—C1—C6—O5	−7.4 (8)
O2—Cd1—N1—C1	−91.4 (4)	N1—C1—C6—O1	−7.5 (8)
O4—Cd1—N1—C1	84.3 (4)	C2—C1—C6—O1	171.2 (5)
O3—Cd1—N1—C1	175.8 (4)	C10—N3—C7—C8	−1.1 (8)
O1—Cd1—N1—C4	−173.7 (5)	Cd1—N3—C7—C8	159.3 (4)
O2—Cd1—N1—C4	99.3 (5)	C9—N4—C8—C7	2.7 (8)
O4—Cd1—N1—C4	−84.9 (5)	C9—N4—C8—C12	−177.8 (5)
O3—Cd1—N1—C4	6.5 (5)	N3—C7—C8—N4	−1.3 (9)
O1—Cd1—N3—C7	96.2 (5)	N3—C7—C8—C12	179.2 (5)
O2—Cd1—N3—C7	−176.9 (5)	C8—N4—C9—C10	−1.8 (9)
O4—Cd1—N3—C7	8.2 (5)	C7—N3—C10—C9	1.9 (8)
O3—Cd1—N3—C7	−83.5 (5)	Cd1—N3—C10—C9	−161.4 (5)
O1—Cd1—N3—C10	−102.6 (4)	C7—N3—C10—C11	−177.0 (5)
O2—Cd1—N3—C10	−15.7 (4)	Cd1—N3—C10—C11	19.7 (6)
O4—Cd1—N3—C10	169.4 (4)	N4—C9—C10—N3	−0.5 (9)
O3—Cd1—N3—C10	77.7 (4)	N4—C9—C10—C11	178.4 (5)
C4—N1—C1—C2	−0.5 (8)	Cd1—O2—C11—O6	176.5 (5)
Cd1—N1—C1—C2	−171.3 (4)	Cd1—O2—C11—C10	−3.6 (7)
C4—N1—C1—C6	178.3 (5)	N3—C10—C11—O6	168.1 (5)
Cd1—N1—C1—C6	7.5 (6)	C9—C10—C11—O6	−10.8 (9)
C3—N2—C2—C1	−2.7 (8)	N3—C10—C11—O2	−11.8 (8)
N1—C1—C2—N2	2.2 (9)	C9—C10—C11—O2	169.3 (6)

### Hydrogen-bond geometry (Å, °)

**Table:**

*D*—H···*A*	*D*—H	H···*A*	*D*···*A*	*D*—H···*A*
O3—H3A···N4^i^	0.75 (6)	2.12 (6)	2.875 (9)	173 (6)
O3—H3B···O5^ii^	0.73 (10)	2.08 (10)	2.740 (8)	150 (10)
O4—H4A···N2^iii^	0.84 (8)	2.04 (8)	2.852 (8)	164 (8)
O4—H4B···O6^iv^	0.93 (11)	1.99 (10)	2.811 (8)	146 (8)
C4—H4···O5^ii^	0.93	2.29	3.211 (7)	169

Symmetry codes: (i) −*x*+1, −*y*+2, −*z*+2; (ii) *x*, *y*+1, *z*; (iii) −*x*+2, −*y*+1, −*z*+1; (iv) *x*+1, *y*, *z*.

### Table 3 π–π stacking interactions (Å) in the title compound

Notes: α is the dihedral angle (°) between the planes of the rings, *d* is the distance (Å) between the ring centroids and Δ is the displacement (Å) of the centroid of ring 2 relative to the intersection point of the normal to the centroid of ring 1 and the least-squares plane of ring 2.

**Table:**

Ring 1	Ring 2	α	*d*	Δ
N1/C1/C2/N2/C3/C4	(N1/C1/C2/N2/C3/C4)^i^	0	3.730 (3)	1.237
N3/C7/C8/N4/C9/C10	(N3/C7/C8/N4/C9/C10)^ii^	0	3.652 (3)	1.307

Symmetry codes: (i) -x+2, -y+1, -z+1; (ii) -x+1, -y+2, -z+2.
